# The effect of a pedometer-based community walking intervention "Walking for Wellbeing in the West" on physical activity levels and health outcomes: a 12-week randomized controlled trial

**DOI:** 10.1186/1479-5868-5-44

**Published:** 2008-09-05

**Authors:** Graham Baker, Stuart R Gray, Annemarie Wright, Claire Fitzsimons, Myra Nimmo, Ruth Lowry, Nanette Mutrie, the Scottish Physical Activity Research Collaboration (SPARColl)

**Affiliations:** 1 Department of Sport, Culture and the Arts, University of Strathclyde, 76 Southbrae Drive, Glasgow, G13 1PP, UK; 2 Strathclyde Institute of Pharmacy & Biomedical Sciences, University of Strathclyde, The John Arbuthnott Building, 27 Taylor Street, Glasgow, G4 0NR, UK; 3 MRC Social and Public Health Sciences Unit, 4 Lilybank Gardens, Glasgow, G12 8RZ, UK; 4 School of Sport and Exercise Sciences at Loughborough University, Ashby Road, Loughborough, Leicestershire, LE11 3TU, UK; 5 Faculty of Sport, Education & Social Sciences, University of Chichester, Bishop Otter Campus, College Lane, Chichester, West Sussex, PO19 6PE, UK

## Abstract

**Background:**

Recent systematic reviews have suggested that pedometers may be effective motivational tools to promote walking. However, studies tend to be of a relatively short duration, with small clinical based samples. Further research is required to demonstrate their effectiveness in adequately powered, community based studies.

**Objective:**

Using a randomized controlled trial design, this study assessed the impact of a 12-week graduated pedometer-based walking intervention on daily step-counts, self-reported physical activity and health outcomes in a Scottish community sample not meeting current physical activity recommendations.

**Method:**

Sixty-three women and 16 men (49.2 years ± 8.8) were randomly assigned to either an intervention (physical activity consultation and 12-week pedometer-based walking program) or control (no action) group. Measures for step-counts, 7-day physical activity recall, affect, quality of life (*n *= 79), body mass, BMI, % body fat, waist and hip circumference (*n *= 76), systolic/diastolic blood pressure, total cholesterol and HDL cholesterol (*n *= 66) were taken at baseline and week 12. Analyses were performed on an intention to treat basis using 2-way mixed factorial analyses of variance for parametric data and Mann Whitney and Wilcoxon tests for non-parametric data.

**Results:**

Significant increases were found in the intervention group for step-counts (*p *< .001), time spent in leisure walking (*p *= .02) and positive affect (*p *= .027). Significant decreases were found in this group for time spent in weekday (*p *= .003), weekend (*p *= .001) and total sitting (*p *= .001) with no corresponding changes in the control group. No significant changes in any other health outcomes were found in either group. In comparison with the control group at week 12, the intervention group reported a significantly greater number of minutes spent in leisure time (p = .008), occupational (p = .045) and total walking (p = .03), and significantly fewer minutes in time spent in weekend (p = .003) and total sitting (p = .022).

**Conclusion:**

A pedometer-based walking program, incorporating a physical activity consultation, is effective in promoting walking and improving positive affect over 12 weeks in community based individuals. The discussion examines possible explanations for the lack of significant changes in health outcomes. Continued follow-up of this study will examine adherence to the intervention and possible resulting effects on health outcomes.

## Background

Recent position statements have re-affirmed the benefits of an active lifestyle [1,2]. The current physical activity recommendation for adults, aged between 18–65 years, to promote and maintain health is to accumulate at least 30 minutes of moderately intense physical activity on at least five days of the week. Promoting accumulative, lifestyle physical activity is an ideal approach to combat the high levels of inactivity evident in global populations [3,4]. Brisk walking has been suggested as the mode of physical activity most likely to increase physical activity at a population level [5] and is the most commonly reported mode of physical activity amongst adults in many populations [3,6]. It is available to almost all individuals with little risk of injury, is a no cost activity and it can be incorporated into peoples' daily routines [7]. Researchers have identified that self determined brisk walking, even in short bouts of 10 minutes, for 30 minutes a day (including simple everyday walking activities such as walking a dog) produce moderate physical activity at the intensity required to achieve health benefits [8,9].

Walking interventions can be effective in reducing body weight, body mass index (BMI), waist and hip circumference, body fat, blood pressure and the cholesterol:high-density lipoprotein (HDL) ratio [10-16] and may be effective in improving mood, affect [14,17,18] and quality of life [19]. Conversely, some studies have demonstrated that a walking intervention is not sufficient to affect any of these health-related outcomes [20-24]. The reasons for such equivocal results are unclear, therefore determining the potential health benefits that can be achieved through walking is crucial to the public health message.

Whilst several meta-analytical and systematic reviews exist that examine how best to promote physical activity [25,26] there is comparatively limited evidence on the most effective methods to specifically promote walking. A recent systematic review from Ogilvie and colleagues (2007) examined the effectiveness of interventions aimed at increasing walking at both an individual and population level. The review concluded that the strongest evidence exists for tailored interventions that are targeted at individuals most motivated to change. The authors suggested that future studies should also attempt to examine whether walking interventions "*are sufficiently frequent, intense, or sustained to produce measurable outcomes in anthropometric, physiological, biochemical or clinical outcomes*" (Ogilvie et al., 2007 p.1207).

One category of intervention, discussed by Ogilvie and colleagues, was the use of pedometers as an integral component of the intervention. A substantial body of research exists that supports the use of pedometers to measure physical activity [27,28]. A recent systematic review examined the association between pedometer use, physical activity levels and a variety of health related outcomes [29]. The authors concluded that pedometer use was significantly associated with increased physical activity levels and reductions in BMI and systolic blood pressure. In 2006 the National Institute for Health and Clinical Excellence (NICE) in the United Kingdom produced a review of pedometer-based intervention studies between 1990 and 2005 [30]. Due to stringent inclusion criteria, conclusions from this review were drawn from only four studies. Both reviews provide support for the suggestion that pedometers may be useful motivational tools for increasing walking. However, there are several limitations when considering the volume of published studies in this area highlighted by these reviews. Studies were predominantly of short duration (< 12 weeks) and based in the United States of America (USA) with small samples consisting mostly of clinical sub-populations. There is limited evidence regarding their effectiveness in non-clinical samples or in countries other than the USA. Additionally, few studies reported more than one outcome variable of interest. There is a need for cross-cultural, sufficiently powered randomized controlled trials to further examine the effectiveness of pedometers in a community setting.

Evidence from two of the studies included in the NICE review suggests that a support structure, that addresses social and cognitive factors, is required for a pedometer-based intervention to be effective [22,31]. A physical activity consultation, using a theoretically grounded framework, constitutes one method of addressing these factors that has been demonstrated to effectively promote physical activity [26,32]. Researchers have suggested that pedometers may provide an important point of discussion for in-depth physical activity consultations [33] yet this is an area that has not been particularly well addressed by pedometer-based intervention studies.

The Walking for Well-being in the West (WWW) study is a multi-dimensional community-based randomized controlled trial which aims to promote and maintain increased walking behavior through the use of physical activity consultations and a pedometer-based walking program. The purpose of this article is to investigate the short term effects of a pedometer based intervention, in conjunction with a physical activity consultation, on walking behavior and health related outcomes in individuals not meeting current physical activity guidelines. Full details of the study design and rationale are reported elsewhere [34] Details of the subsequent stages of the WWW intervention study can be found on the Scottish Physical Activity Research Collaboration website .

## Methods

### Recruitment

The WWW intervention was set in the surrounding community of a West of Scotland university. Recruitment was targeted specifically at individuals in the lowest socio-economic groups. Recruitment was targeted at data zones within 1.5 km of the university campus that were ranked within the top 15% of the Scottish Index of Multiple Deprivation (SIMD) (i.e. the most deprived zones). The SIMD is the official measure of relative area based deprivation in Scotland and is based on 37 deprivation indicators across 7 domains: current income, employment, housing, health, education, skills and training, and geographical access to services and telecommunications. These measures are used to split the country into data zones of between 500 and 1000 people, which are then ranked from the most deprived (1) to least deprived (6505) on the overall SIMD index. The sampling frame of 1.5 km was utilised as it was estimated that this would provide a sufficient number of participants who were within a suitable walking distance from the university campus for assessments. Recruitment in the top 15% of the SIMD index produced few responses and so recruitment was extended to include all households within the specified study area regardless of socio-economic status. Participants were recruited between August and December of 2006 using mail drops, adverts in a local newspaper, posters (placed in physicians' surgeries and shops within the study area) and community stalls.

### Participants

Individuals interested in participating in the study contacted the research team via email, telephone or postal method and in turn were provided with further study information through the individual's preferred mode of contact. Upon satisfactory inspection of this information individuals attended a screening meeting at the research centre to determine suitability for participation. Inclusion criteria were: independently ambulatory, English speaking and between the ages of 18–65 years. Only individuals who were self-classified as not meeting current physical activity recommendations, by means of an adapted stage of exercise behavior change model [35], were invited to participate. This method of screening physical activity behavior was chosen due to the uncertainty of how to determine meeting current recommendations based on the main outcome measure of pedometer step-counts. Classification by stage of change has been shown to be associated with several positive health habits such as fewer health related costs and physical activity stage of change was found to be behaviorally valid as evidenced by self-reported physical activity, self-reported exercise, self-reported sedentary behaviors, pedometers and physical functioning [36]. Participants were also required to complete the Physical Activity Readiness Questionnaire to assess their suitability for an exercise program [37]. A positive response in this questionnaire required a letter of approval from the participant's physician in order to take part. Participants not meeting any aspect of these criteria were excluded from participation and provided with written information on the benefits of physical activity. Written informed consent was obtained from all participants for their involvement in the physical activity intervention. Participants provided additional consent for the optional health related outcome measures. Upon completion of this initial screening meeting participants completed baseline outcome measures and were subsequently randomized. All research procedures were approved by the relevant university research ethics committee. Data for this stage of the study were collected between August 2006 and December 2006.

### Randomization Procedure

The WWW study is a two group (intervention and control) by six time points (baseline, 12, 24, 36, 48 and 60 week) randomized controlled trial. The data reported in this paper concerns the initial intervention stage from baseline to 12 weeks. Randomization was carried out via an independent interactive voice response system (IVRS) which concealed all details of the randomization method from the end users. The IVRS is an interactive telephone based system which allows an authenticated caller (researchers) to randomize a subject into the study. Randomization was stratified by gender (male/female) and baseline step counts (≤ 7,999/≥ 8000) creating a total of four distinct stratification groups. Researchers who conducted the physical activity consultations were not blinded to group assignment in order to implement the physical activity intervention. Additional researchers who performed physiological measurements were blinded to group assignment. The value of 8,000 steps was used as a stratification variable to account for individuals with a high baseline step-count. This value has previously been used as a baseline descriptor for sedentarism [38]. Researchers have also suggested that individuals are more likely to attain public health guidelines by walking at least 8000 steps/day [39]. Positive effects on conventional metabolic parameters, such as blood pressure, have been found when steps are above 8000 steps/day [40].

### Outcome measures

#### Physical Activity

Daily physical activity was measured using two methods. The primary outcome measure was steps/day measured by the Omron HJ-109E Step-O-Meter (Omron Healthcare UK Ltd). To the authors' knowledge this model of pedometer has not been utilized in any previous intervention studies. However, Ryan and colleagues have demonstrated good inter-reliability between units and acceptable accuracy (less than 5% error) at speeds above 1.56 m/s for this model [41]. The Omron HJ-109E has several features beneficial to an intervention of this nature, including a cover to prevent accidental resetting and a 7-day memory which negates the need for participants to record their own step-counts. Daily physical activity was also measured using a 7-day recall of physical activity in the form of the long (self-reported) version of the International Physical Activity Questionnaire (IPAQ) [42]. This subjective measure of physical activity was used to examine changes in modes of physical activity that would not be measured by the pedometer such as swimming or other forms of structured sport or exercise. The IPAQ long version is a 31-item instrument that collects information about moderate and vigorous physical activity across four domains: work-related, transportation, housework/gardening, and leisure time physical activity. This detail on specific physical activity domains therefore allows researchers to identify where changes in physical activity may have occurred. Walking time is also included for the work, transport and leisure domains. Two additional questions measure time spent sitting which can be used as an indication of sedentary time.

#### Health Related Outcomes

Affect (an individuals feelings and emotions) was assessed using the Positive and Negative Affect Schedule (PANAS) [43] which has been demonstrated to be a valid and reliable measure of the constructs in a non-clinical sample of U.K. adults [44]. The PANAS is a self report measure consisting of 10 words relating to positive feelings and emotions, such as 'interested' and 'alert', and 10 words that relate to negative feelings such as 'distressed' and 'upset'. Participants are asked to rate each item according to what extent they have felt that way in the previous few weeks using a Likert scale from (1) very slightly or not at all to (5) extremely. Items were summed to give mean scores (out of 50) for positive affect and negative affect.

Quality of life was measured using the Euroqol EQ-5D instrument [45]. The EQ-5D is a self-report questionnaire comprised of the EQ-5D descriptive system and the EQ VAS. The EQ-5D descriptive system is a five item questionnaire that assesses participants' current health state over five dimensions: mobility; self-care; usual activities; pain/discomfort; and anxiety/depression. Each item is comprised of three levels (no problems, some/moderate problems, extreme problems). A unique health state can be obtained by combining the participants' levels from the five dimensions. The score can then be converted into a weighted health index using "value sets" gained from population data. The UK value set, developed by Dolan and colleagues, was used in this study [46]. The EQ VAS records an individual's self-rated health status on a vertical graduated (0 to 100) visual analogue scale.

Body mass was measured on a precision balance (Sartorius, AG Gottingen, accuracy ± 0.001 kg). From these measurements body mass index (BMI) was calculated as height(m)/weight(kg)^2^; height was measured using a standard laboratory stadometer. Waist-to-hip ratio was calculated from measurements made using a SECA 200 (SECA, Birmingham, UK) measuring tape. Percentage body fat was estimated from skinfold thickness (Harpenden, British Indicators, West Sussex, UK) measurements taken at four sites (biceps, triceps, subscapularis and suprailiac) according to the methods of Durnin and Womersley [47].

Blood pressure was measured using an automated blood pressure monitor (Omron HEM-907, Bannockburn, IL). On each visit blood pressure measurements were performed three times with a rest period of one minute between measurements. Three measurements of resting heart rate were also recorded simultaneously by the blood pressure monitor. The average of these measurements is reported in these results.

Fasting blood samples were taken from an intravenous butterfly cannula inserted into an antecubital vein. Samples were drawn into K^+^EDTA vacutainers (BD Biosciences, Oxford, UK) which were centrifuged and the plasma removed and stored at -80°C for subsequent analysis. Total cholesterol and high-density lipoprotein (HDL) cholesterol (direct method), from the plasma, were measured in duplicate on a fully automated spectrophotometric analyzer (Pentra 400, Horiba-ABX, Montpellier, France) using commercially available kits (Horiba-ABX, Montpellier, France). The co-efficient of variation for these assays was as follows: total cholesterol: 1.4%, HDL-cholesterol: 1.6%.

### Procedures

All participants completed a baseline week wearing a pedometer, sealed with tape, for seven days at all times (except when showering, sleeping or taking part in structured sport or exercise) with instructions not to alter their daily routine. The WWW study was designed to impact walking behavior therefore only ambulatory activity was recorded. Irregular bouts of structured sport or exercise that could significantly affect an individual's mean step-count were not recorded. Each pedometer was individually calibrated consistent with manufacturers guidelines to within 5% of actual steps walked in a 100-step test. Participants were required to provide at least five days of step counts including at least one weekend day to gain an accurate reflection of physical activity levels. Pre-intervention measures of all health related outcomes were obtained and participants completed the IPAQ before being randomly assigned to either intervention or control group.

Participants assigned to the intervention group received a physical activity consultation and then followed a 12-week pedometer-based walking program. The sessions were based on a theoretical framework, recommended to ensure quality [48]. The Transtheoretical Model of exercise behavior change (TTM) [49] was chosen for this purpose as there are published guidelines for health professionals to conduct consultations using this model as a theoretical framework [50,51]. The TTM is a common theoretical framework for physical activity consultations [52] and has been used successfully in intervention studies designed to increase physical activity in a Scottish population [32,53,54].

The consultations were semi-structured following established guidelines [50,51]. A guiding style was used with participants making decisions about how to change their walking behavior [55]. The consultations were focused on the uptake of physical activity, in this context promoting increases in walking. Strategies used included enhancing motivation, overcoming barriers and developing appropriate walking plans which were tailored to the individual as recommended by Ogilvie and colleagues (2007). The sessions also included discussion of the three mediators of the TTM that have been shown to be important to behavior change [49]. These are self-efficacy (confidence in ability to change), decisional balance (pros and cons of change) and processes of change (strategies and techniques used to change, e.g., social support). Although the sessions were flexible and individualized, the TTM was used to create a standard protocol to follow during the sessions. Table 1 shows the steps followed.

**Table 1:** Key points covered during physical activity consultations

	Key points covered
Step 1: Physical Activity History	• Participants' reasons for increasing walking
	• Consider why walking is attractive to them
	• Current walking levels
	
Step 2: Decisional Balance	• Weigh up pros and cons of increasing walking
	• Minimize any perceived cons
	
Step 3: Barriers	• Consider barriers to increasing walking
	• Consider how to overcome these barriers
	
Step 4: Goal-setting	• Explanation of walking program and pedometer
	• Informed of baseline step-counts
	• Discuss realistic and time-phased goals
	• Identify situations for increasing walking
	• Identify local walking routes
	
Step 5: Summarize	• Check self-efficacy of achieving goals

The walking program was based on a 12-week time frame: the first six weeks consisted of graduated bi-monthly goals with an aim for the increased walking behavior to be maintained for the remaining six weeks. The overall goal of the walking program was for participants to increase their mean daily step-count by 3,000 accumulated steps above their baseline value on five days of the week. This value is based on the assumption that moderate brisk walking produces 100 steps a minute (1,000 steps per 10 minutes) [56] therefore 3,000 steps would equate to approximately 30 minutes of moderate physical activity, in line with current physical activity recommendations [57]. This program has previously been successfully used with similar UK samples over a shorter time-frame [58]. Intervention group participants retained their pedometers, open for feedback, for the duration of the intervention period and were shown how to use this to monitor their daily step-counts. The full list of goals is displayed in Table 2. Goals were retained for two consecutive weeks to enable participants to reinforce their increased levels of walking, or to try other strategies to successfully accumulate the additional steps. Participants were advised on the nature of the intensity and duration of the desired increases in walking. Participants were familiarized with the Borg 6–20 scale [59] and advised that additional walking should be of a brisk nature that would leave them slightly breathless and hot but still able to talk (indicated as between 12–14 on this scale). Bouts of at least 10 minutes in duration were advised for additional walking although the accumulation of walking during everyday tasks wherever possible was also advocated.

**Table 2:** Weekly goals of intervention group participants

**Time-point**	**Goal**
Week 1	To walk an extra 1,500 steps (from baseline value) on at least 3 days of the week
Week 2	To walk an extra 1,500 steps (from baseline value) on at least 3 days of the week
Week 3	To walk an extra 1,500 steps (from baseline value) on at least 5 days of the week
Week 4	To walk an extra 1,500 steps (from baseline value) on at least 5 days of the week
Week 5	To walk an extra 3,000 steps (from baseline value) on at least 3 days of the week
Week 6	To walk an extra 3,000 steps (from baseline value) on at least 3 days of the week
Week 7	To walk an extra 3,000 steps (from baseline value) on at least 5 days of the week
Week 8	To walk an extra 3,000 steps (from baseline value) on at least 5 days of the week
Weeks 9–12	To maintain walking levels using the week 7 goal

Participants assigned to the control group were asked to maintain their normal walking levels between baseline and week 12. At the end of week 11 these participants collected an individually calibrated pedometer from the research centre and wore this sealed during week 12 to gain a record of their step-counts. At the end of week 12 researchers met with all participants to record step-counts at which time participants completed the IPAQ and post-intervention health measures were taken.

### Statistical Power and Analyses

G-Power analysis [60] set for F-test analysis of variance (ANOVA) was used to calculate sample size for between group analyses of the primary outcome measure (i.e., daily step count). Power was set at 0.8, Alpha level was set at 0.05 and effect size (Cohen's *f *[61]) was set at 0.4 (large) for the two group (intervention and control) design based on previous unpublished work from this research group. A minimum sample size of 52 was calculated (26 participants in each group).

Data were analyzed using SPSS v.14.0 (SPSS, Inc, Chicago, IL). All results reported were analyzed by the main intervention groups: analyses by separate stratification variables (gender, baseline step-count) found no significant interaction effects between groups (males/females and ≤ 7,999/≥ 8000) therefore are not presented here. The analyses were performed on an intention to treat basis. Missing week 12 data (due to participant drop-out) were substituted with the participants' baseline value. Baseline differences between the intervention and control group were examined using independent t-tests. Steps/day and health related outcome data were analyzed using two-way mixed factorial analyses of variance (ANOVA). Missing weekday step-count data were replaced by inputting the mean of the remaining weekdays and missing weekend step-count data were replaced by inputting the alternate weekend day [62]. Exploratory analysis revealed that data from several sub-sections of the IPAQ were non-normally distributed. Non-parametric analyses were therefore used to analyze these data. Mann Whitney U tests were used to examine between group differences and Wilcoxon's signed-rank tests were used to examine within group differences over time. Due to the number of variables available from the IPAQ only statistically significant results are presented. Statistical significance was defined as *p *< 0.05 for all tests with data presented as mean (SD) unless otherwise stated.

## Results

### Participants

**Figure 1:**
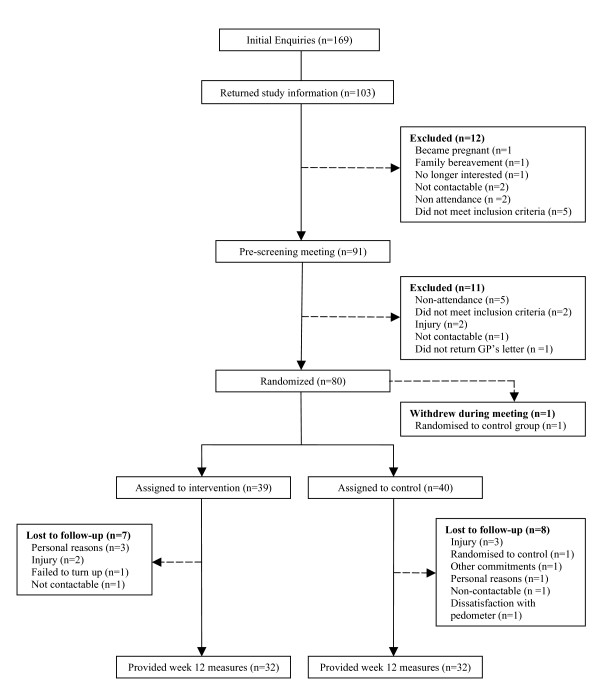
Flow of participants through the study.

From 169 initial enquiries to the study, 91 individuals met the inclusion criteria and provided informed consent at an initial meeting. Seventy-nine participants (63 females, 16 males, age = 49.2 ± 8.9) provided baseline measurements of pedometer step counts, IPAQ and measures of affect and quality of life (specific numbers for participants who consented to other health related outcome measures can be found in Table 4). The intervention group (*n *= 39) consisted of 31 females and eight males and the control group (*n *= 40) consisted of 32 females and eight males. Overall, 55 of 79 participants (70%) were below the randomization stratification variable of 8,000 steps at baseline: this consisted of 28 of 39 (72%) of participants in the intervention group and 27 of 40 (68%) of participants in the control group. Figure 1 displays the flow of participants through the study. As shown in Figure 1 there were 15 participants who withdrew from the study between baseline and week 12. The following results are presented on an intention to treat basis where all participants were considered. Table 3 displays the proportion of participants in each level of the five dimensions in the EQ-5D descriptive system. Table 4 displays descriptive statistics (mean [*M*] and standard deviation [*SD*]) for age, pedometer steps and all health related outcomes, at baseline and week 12. Table 5 displays descriptive statistics (median [*Mdn*] and range [*r*] for all IPAQ variables at baseline and week 12.

**Table 3:** Number of participants in each level for the five domains of the EuroQol EQ-5D descriptive system

		Mobility		Self Care		Usual Care		Pain/Discomfort		Anxiety/Depression	
		Baseline	Week 12	Baseline	Week 12	Baseline	Week 12	Baseline	Week 12	Baseline	Week 12
						
No Problems	Intervention^a^	34	33	39	39	35	34	27	30	25	24
	Control^b^	33	33	40	40	35	35	26	28	28	27
Some/Moderate	Intervention	5	6	0	0	4	5	12	9	14	15
	Control	7	7	0	0	5	5	14	12	12	13
Extreme Problems	Intervention	0	0	0	0	0	0	0	0	0	0
	Control	0	0	0	0	0	0	0	0	0	0

^a ^intervention group (n = 39).

^b ^control group (n = 40)

**Table 4:** Descriptive statistics for age, pedometer step-counts and health related outcomes at baseline and week 12 for intervention and control group. Values are mean (*M*) and standard deviation (*SD*).

	Intervention Group (n = 39)		Control Group (n = 40)	
	Baseline	Week 12	Baseline	Week 12
	
Age (years)	47.3 (9.3)	^a^	51.2 (7.9)	^a^
Steps/day	6802 (3212)	9977 (4669)	6924 (3201)	7078 (2911)
PANAS positive	31.2 (6.7)	33.5 (7.4)	31.7 (6.9)	31.3 (7.6)
PANAS negative	20.1 (7.2)	19.1 (6.9)	20.2 (8.1)	18.8 (7.5)
EQ-5D tariff	0.88 (0.12)	0.89 (0.11)	0.87 (0.12)	0.89 (0.12)
EQ VAS	65.4 (18.3)	69.5 (17.8)	69.8 (19.7)	70.7 (18.6)
Height (m)^b^	1.66 (0.08)	^a^	1.64 (0.08)	^a^
Body Mass (kg)^b^	78.9 (15.6)	79.1 (15.2)	79.5 (18.1)	79.6 (17.8)
BMI (kg/m^2^)^b^	28.5 (4.8)	28.7 (4.8)	29.4 (6.3)	29.5 (6.2)
Waist circumference (cm)^b^	89.5 (12.6)	89.9 (12.6)	90.4 (14.6)	91.1 (15.6)
Hip circumference (cm)^b^	108.9 (8.8)	108.6 (9.7)	110.1 (12.4)	110.3 (11.8)
Waist:Hip Ratio^b^	0.82 (0.08)	0.83 (0.08)	0.82 (0.09)	0.82 (0.09)
% body fat^b^	30.7 (4.4)	31. (4.9)	31.8 (5.6)	32.7 (6.3)
Systolic blood pressure (mm Hg)^b^	118.2 (17.9)	119.6 (17.0)	119.9 (15.9)	121.9 (15.1)
Diastolic blood pressure (mm Hg)^b^	75.1 (11.4)	77.1 (12.1)	75.5 (11.8)	79.1 (11.6)
Heart Rate (beats.min^-1^)^c^	68.6 (7.2)	69.8 (7.2)	67.9 (8.6)	69.2 (9.0)
Total Cholesterol (mmol.l^-1^)^c^	5.4 (1.3)	5.4 (1.2)	5.5 (1.1)	5.5 (1.0)
HDL (mmol^-1^)^c^	1.3 (0.3) 50.7	1.3 (0.3)	1.4 (0.4) 54.6	1.4 (0.4)
Chol:HDL Ratio^c^	4.2 (1.1)	4.2 (1.1)	4.1 (1.2)	4.0 (1.3)

^a ^not measured at week 12.

^b ^anthropometric measures: (n = 37) for intervention group, (n = 39) for control group.

^c ^blood measures: (n = 32) for intervention group, (n = 34) for control group.

Note: there were no significant differences between the intervention and control group for any variable at baseline.

**Table 5:** Descriptive statistics for IPAQ variables at baseline and week 12. Values are median (*Mdn*) and range (*r*).

	Intervention Group (n = 39)		Control Group (n = 40)	
	Baseline	Week 12	Baseline	Week 12
	
**Work-related PA**				
Vigorous PA	0 (1080)	0 (1800)	0 (720)	0 (540)
Moderate PA	0 (1500)	0 (900)	0 (1500)	0 (600)
Walking	0 (1620)	0 (2520)	0 (1350)	0 (1650)
Total	0 (3000)	0 (4680)	0 (2500)	0 (2730)
				
**Transportation PA**				
Bicycling	0 (0)	0 (0)	0 (40)	0 (0)
Walking	105 (1680)	90 (900)	80 (1680)	40 (840)
Total	105 (1680)	90 (900)	80 (1720)	40 (840)
				
**Housework PA**				
Vigorous outside home	0 (840)	0 (120)	0 (750)	0 (360)
Moderate outside home	0 (2100)	0 (1680)	0 (1260)	0 (720)
Moderate inside home	210 (2100)	112.5 (840)	180 (1680)	90 (1260)
Total	360 (4200)	120 (2520)	255 (2640)	145 (2100)
				
**Leisure-time PA**				
Walking	40 (840)	100 (840)	35 (600)	16.25 (840)
Vigorous PA	0 (180)	0 (120)	0 (180)	0 (180)
Moderate PA	0 (360)	0 (60)	0 (120)	0 (600)
Total	60 (840)	90 (840)	60 (600)	11 (840)
				
**Combined Domains**				
Total Walking	225 (3360)	260 (2850)	167.5 (1740)	90 (1925)
Total Moderate PA	420 (4380)	120 (2760)	360 (2640)	175 (2100)
Total Vigorous PA	0 (1080)	0 (1800)	0 (720)	0 (600)
Total PA	690 (6300)	590 (5415)	640 (4300)	500 (3185)
				
**Time Spent Sitting**				
Weekday	1500 (3750)	1200 (3900)	1500 (3450)	1500 (2850)
Weekend	480 (1320)	360 (1200)	600 (1200)	600 (1320)
Total	2265 (4650)	1680 (5100)	2130 (4170)	2100 (3630)

### Physical Activity

#### Step counts

**Figure 2:**
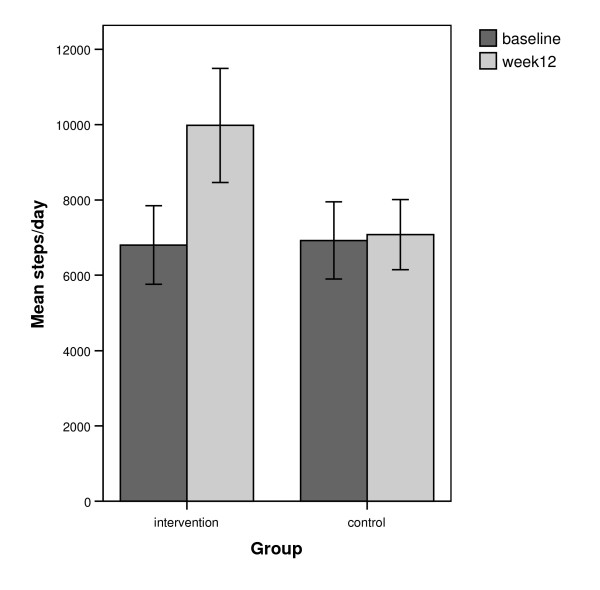
Mean steps/day for intervention group (n = 39) and control group (n = 40) at baseline and week 12.

Figure 2 displays the mean steps/day for both groups at both time-points. A significant interaction was identified between group (intervention, control) and time (baseline, week 12) in terms of the recorded step-counts, (*F*_(1,77) _= 25.18, *p *< .001, partial η^2 ^0.25). A paired t-test found a significant increase in steps/day for the intervention group between baseline (*M *= 6802, *SD *= 3212) and week 12 (*M *= 9977, *SD *= 4669, *t*(38) = -6.06, *p *< .001, *d *= 0.79, confidence intervals [*CI*] 2,115 – 4236). No significant difference was observed in the control group between baseline (*M *= 6924, *SD *= 3201) and week 12 (*M *= 7078, *SD *2911, *t*(39) = -0.50, *p *= 0.618, *CI *-463 – 770). The mean difference in change between the two groups was 3,022 steps/day and was statistically significant (*t*(77) = 5.02, *p *< .001, *d *= 1.96). Chi-square analysis determined that a significantly greater percentage (χ^2 ^= 24.88, *p *< .001) of participants in the intervention group (25/39, 64%) achieved an increase of 15,000 steps per week, equivalent to physical activity guidelines of the accumulation of 150 minutes of moderate physical activity, compared with the control group (4/40, 10%).

#### 7-day recall of physical activity (IPAQ)

Wilcoxon's signed-rank tests revealed that at week 12 the intervention group recalled a significant increase in the number of leisure minutes walked (*Z *= 2.32, *p *= 0.02, *r *= 0.37, median [*Mdn*] difference = 100 minutes per week) and a significant decrease in weekday sitting (*Z *= 2.94, *p *= 0.003, *r *= 0.47, *Mdn *difference = 1200 minutes per week), weekend sitting (*Z *= 3.41, *p *= 0.001, *r *= 0.55, *Mdn *difference 360 minutes per week) and total sitting (*Z *= 3.38, *p *= 0.001, *r *= 0.54, *Mdn *difference = 1680 minutes per week) from baseline. At week 12 the control group recalled a significantly greater number of vigorous leisure minutes of physical activity (Z = 2.02, p = 0.043, *r *= 0.32, *Mdn *difference = 0 minutes) than at baseline. This result is due to five individuals in the control group increasing their vigorous leisure minutes recalled. As the majority of participants (34 of 40) report zero minutes at both time-points the median difference equals zero despite the group reporting a significant increase.

Mann Whitney U tests revealed that at week 12 the intervention group recalled a significantly greater number of leisure minutes walked (*U *= 513.00, *p *= 0.008, *r *= 0.30, *Mdn *difference 83.8 minutes), number of occupational minutes walked (*U *= 602.00, *p *= 0.045, *r *= 0.23, *Mdn *difference 0 minutes) and total number of minutes walked (*U *= 560.50, *p *= 0.03, *r *= 0.24, *Mdn *difference = 57.5 minutes) than the control group. The intervention group also recalled significantly less total time spent sitting (*U *= 546.00, *p *= 0.022, *r *= 0.26, *Mdn *difference = -420 minutes) due to significantly less time spent sitting at the weekend (*U *= 474.50, *p *= 0.003, *r *= 0.34, *Mdn *difference = -240 minutes).

### Health related outcomes

#### Affect (PANAS)

A significant interaction was identified between group (intervention, control) and time (baseline, week 12) in terms of the positive affect scores, (*F*_(1,77) _= 4.26, *p *= .042, partial η^2 ^0.05). A paired t-test found a significant increase in positive affect for the intervention group between baseline (*M *= 31.2, *SD *= 6.7) and week 12 (*M *= 33.5, *SD *= 7.4, *t*(38) = 2.29, *p *= .027, *d *= *0.33*, *CI *.27 – 4.39). No significant difference was observed in the control group between baseline (*M *= 31.7, *SD *= 6.9) and week 12 (*M *= 31.3, *SD *7.6, *t*(39) = -0.524, *p *= 0.604, -2.31 – 1.36). There was no significant interaction or main effect found for the negative affect scores or for any of the other health related outcomes measured in the present study (Table 4).

## Discussion

This study is one of the first adequately powered, UK based randomized controlled trials to examine the impact of a pedometer-based walking intervention on step-counts in a community setting. The major finding of this study was that a graduated pedometer-based walking program, in conjunction with a physical activity consultation increased walking in low-active adults over a period of 12 weeks. The control group, included to account for the intrinsic motivation of volunteer participants [63], displayed no significant change in steps/day over time. The conservative intention to treat analysis (baseline carried forward for missing values) produced a mean change in the intervention group of 3,175 steps/day, an increase of 47% above baseline values, a favorable increase compared with other pedometer-based randomized controlled trials [22,31,33]. In their systematic review, Bravata and colleagues (2007) reported that pedometer users increase physical activity by an average of 26.9% over baseline values.

The overall goal for participants was to increase their baseline step-counts by 3,000 steps/day on five days of the week (i.e., equivalent to current physical activity recommendations); equating to an overall weekly increase of 15,000 steps/week. The intervention group displayed a mean increase in weekly step counts of 22,225 steps/week thus exceeding the recommended goal. At an individual level 64% of participants in the intervention group achieved the goal of 15,000 steps/week. Participants in the intervention group progressed from being classified as "low active" (5,000–7,499 steps/day) to within 23 steps of being classed as "active" (≥ 10,000) according to suggested public health ranges for pedometer counts [64]. These results suggest that the intervention is a successful method of promoting walking and allowing individuals to meet suggested public health recommendations.

One issue of the study, also discussed in the Bravata *et al*. systematic review, is that it is not possible to disentangle the respective contributions of the physical activity consultation from the benefits of the pedometer-based goals of the walking program. Physical activity consultations have been demonstrated to be effective at promoting physical activity [32,54], and this particular walking program has previously been shown to be effective at promoting short-term increases in walking [58]. Consistent with recommendations from Bravata and colleagues, the next stage of the WWW project involves the control group following the 12 week walking program without the additional physical activity consultation, while the intervention group will be provided with continued support in the form of a further physical activity consultation. These longer term comparative results will help to determine the most effective components of this intervention.

The increase observed in the step-count data was supported by the self-reported results of the IPAQ; an increase in reported minutes of leisure time walking was found in the intervention group (median increase of 100 minutes per/week). While it is not directly comparable, the increase of 22,225 steps/week observed in the intervention group is approximately equivalent to an additional 222 minutes of walking per week. Although we must consider possible errors in perception when recalling walking [65] this suggests that the primary means by which participants increased their walking was during their leisure time, consistent with previous research [66]. The discrepancy between the objective and subjective measures of activity could be partially explained by the pedometer measuring all activity and the IPAQ only measuring activity in bouts of 10 minutes and greater. Alternatively, additional increases in step-counts could be attributed to an accumulation of walking in other domains and other non-significant increases in ambulatory activity. This may be demonstrated in the significant difference in occupational and total minutes of walking recalled between the intervention and control groups at week 12.

The IPAQ also revealed that the intervention group reduced time spent sitting during both weekdays and weekend days. Researchers have suggested that sedentary behavior, such as time spent sitting, is positively associated with coronary heart disease risk factors, obesity and development of the metabolic syndrome [67-69]. It has been suggested that interventions should seek to both increase physical activity and to decrease sedentary behavior [70]. In this study we did not aim to directly reduce sedentary behavior and as the IPAQ does not measure sitting time across a specific domain it is impossible to determine where the reductions in sitting time occurred. Future interventions incorporating physical activity consultations should ensure that identifying and reducing sedentary behaviors is an integral part of the consultation process and that any physical activity questionnaires used should describe sedentary behaviors to the same extent as physical activity behaviors. These results should be treated with a degree of caution given the disparity between the reduced time spent sitting as indicated by the IPAQ (4 hours of total sitting per day) and the increase in physical activity as measured by the pedometer (approximately 30 minutes of activity per day). It may be possible that non-sitting activities such as standing may attribute to a proportion of this difference however this does not provide an adequate explanation given the magnitude of this observed discrepancy. A more probable explanation is that the error and limitations associated with self-report measures of physical activity [71] are also associated with self-report measures of sedentary behaviors. Preliminary evidence that compares activity levels as measured by a pedometer and the IPAQ would suggest that direct comparisons are unwise as evident by low correlations between the two methods [72].

Outcome measures were only assessed at baseline and week 12. Participants were asked to record their pedometer readings from intermittent sub-goals in self-recorded diaries. Completion of these was not an essential requirement of participants and as such these were often incomplete. The level of missing data in these diaries made it impractical to perform statistical analysis on a week by week basis. We were reluctant to constantly monitor participants to try and mirror a 'real-world' scenario. As a result we were unable to determine whether participants actually achieved, or exceeded, their targets in the periods between assessments. Therefore, the possibility remains that, in a worst case scenario, participants increased their physical activity levels only during week 12 in order to achieve their final target; although such a substantial increase in step-counts over a one week period would be unlikely. A previous study from our research group [58] that followed individuals throughout the intervention reported an incremental increase in step-counts on a week by week basis. While only measuring at pre and post intervention may be seen as a limitation of the current study, this approach removes the possibility of increased motivation through researcher presence and may also decrease the risk of participant drop-out due to repeated meetings. Making completion of step-count diaries a requirement of participants would have increased the level of data available for analysis. However, this substantially increases the responsibility on the participant which, over a prolonged time-period, may provide a potential source for participant drop-out. Conversely, filling in the step-count diaries on a regular basis may act as an extrinsic motivational factor; which could potentially mask the effects of the walking program or the consultation. The recent systematic review on pedometer use suggests that future trials compare pedometer use with versus without a step-count diary [29] and the results of this study support this suggestion given the issues discussed above.

In this study contact with intervention group participants was minimal and yet significant behavior change was still achieved. Following the RE-AIM framework for health behavior interventions [73], this intervention was simple to implement, was efficacious at an individual level and has the capacity to be adopted and implemented within a variety of real-world settings.

Whilst the current study was successful in increasing the daily walking of participants, there was no effect on any of the health-related outcomes measured other than a small to medium, significant increase in positive affect reported by the intervention group. There is contradicting evidence regarding the relationship between walking and affect. Some researchers have suggested that walking may not be performed at a sufficient intensity to produce corresponding positive changes in affect [74]. Conversely, the current study supports research that has demonstrated a positive benefit on affect following a walking intervention [14,18,75]. Researchers have also demonstrated that short, acute bouts of walking may also improve affect [17,76]. These findings provide support for walking as a pleasurable activity and it has been proposed that this enjoyment is linked to intrinsic motivation and subsequent adherence to physical activity [76].

The lack of changes in the other health-related outcomes measured in this study may not be unexpected given the pragmatic approach of the walking intervention. The walking program was designed to increase participants' walking in graduated bi-monthly stages: participants began the program by accumulating an additional 1,500 steps on at least three days of the week for the first two weeks before progressing with the frequency of this goal and then the quantity of walking. This approach was utilized to allow participants the opportunity to follow progressive short term goals in order to reinforce successful strategies or attempt alternative strategies in order to achieve their goals. Subsequently, if this approach was followed by participants, the goal of accumulating an additional 3,000 steps at least five days of the week was only applicable for the final six weeks of the intervention. Recent meta-analyses [13,15], that have found decreases in body weight, BMI, percentage body fat, blood pressure and the cholesterol:HDL ratio after a walking intervention, report means and ranges of duration and frequency consistent with the 30 minutes (3000 steps), five days a week target promoted in the current study. These meta-analyses used data from studies ranging between eight and 104 weeks in length.

It may be the case therefore, that a period longer than six weeks at this duration and frequency is required to allow positive physiological changes to occur. Future follow-up stages of the WWW study will attempt to evaluate this aspect. However, as previously mentioned; we are unable to conclude whether participants strictly followed the graduated approach or whether they were successful in doing so throughout the program. Indeed, previous work investigating the dose-response relationship has shown that it can take up to two years for an increase in HDL to occur as the result of exercise training [77]. On the other hand similar changes in HDL and other anthropometric variables have occurred after 12 weeks in other investigations [16,78]. It is possible, therefore, that the current intervention may have resulted in positive health benefits if it had continued.

Although we may not have expected physiological changes at week 12 due to the graduated walking program, we must consider alternative methodological considerations of the study that may have contributed to the lack of significant changes in health outcomes. With the exception of BMI, participants' health outcomes values were deemed to be within normal ranges at baseline. Significant, and clinically meaningful, changes will consequently be harder to achieve in this population and may indeed be unnecessary for many participants. It has been recommended that 45–60 minutes of moderate physical activity per day is required for weight maintenance [79]. Therefore it was unlikely that these changes would have occurred following an increase of approximately 30 minutes of walking. This study also did not attempt to control for dietary factors. Previous studies have demonstrated that calorific restriction is a more effective weight loss tool than physical activity [80]. As we did not control for or monitor diet it is possible that participants may have compensated for the increase in physical activity by increasing energy intake or by deceasing energy expenditure during other parts of the day. Such a possibility is supported by previous research showing that there is an increase in appetite after a single bout of exercise [81] although this is not always been the case [82]. In the current study no measure of energy intake/expenditure was made hence we are unable to determine whether an increase in energy intake, or decreasing energy expenditure in other parts of the day, has counteracted the increase in energy expenditure due to the walking.

Finally, it may be that the intensity of the walking undertaken by the participants was not sufficient to stimulate health benefits. It is possible that the changes in step-count observed in our participants were caused by short (less than 10 minutes) bouts at low speeds. To date there has been little community based work where the intensity of physical activity has been closely monitored. Results from this and similar community based studies [14,16,23] present equivocal and conflicting findings of producing corresponding physiological changes following successful behavior change. This illustrates that in comparison with controlled, laboratory based studies positive changes in health may be more difficult to achieve in a real world setting, where the frequency, duration and intensity of the intervention cannot be objectively measured.

### Study strengths and limitations

Strengths of this study include using a sealed pedometer at baseline and the use of a pedometer with a 7-day memory which negates the need for participants to record their daily steps. This study is also one of the first UK based, adequately powered randomized controlled trials to examine the effectiveness of pedometers as motivational tools within a community based, non-clinical sample. The use of a multidisciplinary approach to provide measures of health related outcomes adds significantly to the study, and it is of considerable importance that all analyses were performed on an intention to treat basis. The lack of an intention to treat approach in the literature has been identified as a weakness when considering application to a population setting [15].

Although the pedometer is a useful measurement tool with regards to ambulatory physical activity, one limitation of the instrument is that the lack of any direct measure of intensity makes it difficult to draw conclusions about time spent in moderate or vigorous physical activity. Pedometers are currently being developed that address the issue of measuring only walking at a pre-determined moderate intensity (aerobic steps) although further research into the validity of these measures is required before they can be used confidently in intervention studies. The pedometer measures ambulatory activity, not strictly walking, which suggests that the increase in steps/day found between baseline and week 12 in the intervention group could be attributed to other forms of physical activity. However, participants were asked to remove the pedometer whilst engaging in structured sport or exercise, and self-report measures of physical activity do not show evidence of changes in ambulatory activity other than walking.

## Conclusion

In summary, this study has demonstrated that a 12-week pedometer-based walking program in combination with a physical activity consultation was an effective way to increase walking, reduce sedentary behavior and increase positive affect in a community based sample not meeting current physical activity recommendations. The intervention was relatively simple to implement and has the capacity to be reproduced in a variety of settings. The intervention was not sufficient to induce beneficial physiological changes. Future stages of this study will examine adherence to the intervention, compare the effects of the walking program with versus without the physical activity consultation and provide longitudinal data on health related outcomes.

## Abbreviations

NICE: National Institute for Health and Clinical Excellence; WWW: Walking for Well-being in the West; IPAQ: International Physical Activity Questionnaire; PANAS: Positive and Negative Affect Schedule; BMI: Body mass index; HDL: High-density lipoprotein; TTM: Transtheoretical Model of exercise behavior change; ANOVA: Analysis of Variance; M: Mean; SD: Standard deviation; Mdn: Median.

## Competing interests

The authors declare that they have no competing interests.

## Authors' contributions

GB participated in the design and coordination of the study, was responsible for data collection and statistical analysis on step-counts, IPAQ, mood and quality of life measures, performed the physical activity consultations and drafted the manuscript. SG was responsible for collection of health related outcome data, assisted with drafting the health related outcome sections of the manuscript, performed statistical analysis on the health related outcome measures and provided feedback during the manuscript preparation stage. AW participated in the design and coordination of the study, was responsible for data collection of step-counts, IPAQ, mood and quality of life measures, performed the physical activity consultations and provided feedback during the manuscript preparation stage. CF participated in the design and coordination of the study, assisted with collection of the health related outcome data and provided feedback during the manuscript preparation stage. MN participated in the design of the study, assisted with collection of the health related outcome data and provided feedback during the manuscript preparation stage. RL participated in the design of the study, statistical analysis and provided feedback during the manuscript preparation stage. NM (on behalf of SPARColl) was principal investigator, conceived the study, participated in the design of the study and provided feedback during the manuscript preparation stage. All authors read and approved the final manuscript.
