# The effect of a pedometer-based community walking intervention "Walking for Wellbeing in the West" on physical activity levels and health outcomes: a 12-week randomized controlled trial

**DOI:** 10.1186/1479-5868-7-51

**Published:** 2010-05-27

**Authors:** Graham Baker, Stuart R Gray, Annemarie Wright, Claire Fitzsimons, Myra Nimmo, Ruth Lowry, Nanette Mutrie

**Affiliations:** 1 Medical Research Council Social and Public Health Sciences Unit, 4 Lilybank Gardens, Glasgow, G12 8RZ, Scotland

## Correction

Since publication of our article[1] we have found some imputation errors in the main text and Table Four (Table 1) and Table Five (Table 2) corresponding to the 12-week results. The corrections are as follows:

**Table 1:** Descriptive statistics for age, pedometer step-counts and health related outcomes at baseline and week 12 for intervention and control group.

	Intervention Group (n = 39)		Control Group (n = 40)	
	**Baseline**	**Week 12**	**Baseline**	**Week 12**

Age (years)	47.3 (9.3)	^a^	51.2 (7.9)	^a^

Steps/day	6802 (3212)	9977 (4669)	6924 (3201)	7078 (2911)

PANAS positive	31.2 (6.7)	33.5 (7.4)	31.7 (6.9)	31.3 (7.6)

PANAS negative	20.1 (7.2)	19.1 (6.9)	20.2 (8.1)	18.8 (7.5)

EQ-5D tariff	0.88 (0.12)	0.89 (0.11)	0.87 (0.12)	0.89 (0.12)

EQ VAS	65.4 (18.3)	69.5 (17.8)	69.8 (19.7)	70.7 (18.6)

Height (m)^b^	1.66 (0.08)	^a^	1.64 (0.08)	^a^

Body Mass (kg)^b^	78.9 (15.6)	79.1 (15.2)	79.5 (18.1)	79.5 (17.8)

BMI (kg/m^2^)^b^	28.5 (4.8)	28.6 (4.8)	29.4 (6.3)	29.5 (6.2)

Waist circumference (cm)^b^	89.5 (12.6)	89.8 (12.7)	90.4 (14.6)	90.9 (15.6)

Hip circumference (cm)^b^	108.9 (8.8)	108.5 (9.7)	110.1 (12.4)	110.2 (11.8)

Waist:Hip Ratio^b^	0.82 (0.08)	0.83 (0.08)	0.82 (0.09)	0.82 (0.09)

% body fat^b^	30.7 (4.4)	31.2 (4.9)	31.8 (5.6)	32.5 (6.3)

Systolic blood pressure (mm Hg)^b^	118.2 (17.9)	119.2 (17.0)	119.9 (15.9)	121.4 (15.1)

Diastolic blood pressure (mm Hg)^b^	75.1 (11.4)	76.3 (12.2)	75.5 (11.8)	78.2 (11.8)

Heart Rate (beats.min^-1^)^c^	68.6 (7.2)	69.5 (8.3)	67.9 (8.6)	68.9 (9.0)

Total Cholesterol (mmol.l^-1^)^c^	5.4 (1.3)	5.4 (1.2)	5.5 (1.1)	5.5 (1.0)

HDL (mmol^-1^)^c^	1.3 (0.3)	1.3 (0.3)	1.4 (0.4)	1.4 (0.4)

Chol:HDL Ratio^c^	4.2 (1.1)	4.2 (1.1)	4.1 (1.2)	4.1 (1.3)

Values are mean (*M*) and standard deviation (*SD*).

^a ^not measured at week 12

^b ^anthropometric measures: (n = 37) for intervention group, (n = 39) for control group

^c ^blood measures: (n = 32) for intervention group, (n = 34) for control group

Note: there were no significant differences between the intervention and control group for any variable at baseline

**Table 2:** Descriptive statistics for IPAQ variables at baseline and week 12.

	Intervention Group (n = 39)		Control Group (n = 40)	
	**Baseline**	**Week 12**	**Baseline**	**Week 12**
	
**Work-related PA**				
Vigorous PA	0 (1080)	0 (1800)	0 (720)	0 (540)
Moderate PA	0 (1500)	0 (900)	0 (1500)	0 (600)
Walking	0 (1620)	0 (2520)	0 (1350)	0 (1650)
Total	0 (3000)	30 (4680)	0 (2550)	0 (2730)
				
**Transportation PA**				
Bicycling	0 (0)	0 (0)	0 (40)	0 (40)
Walking	105 (1680)	140 (900)	80 (1680)	70 (1680)
Total	105 (1680)	140 (900)	80 (1720)	70 (1720)
				
**Housework PA**				
Vigorous outside home	0 (840)	0 (840)	0 (750)	0 (360)
Moderate outside home	0 (2100)	0 (1680)	0 (1260)	0 (840)
Moderate inside home	210 (2100)	150 (840)	180 (1680)	120 (1680)
Total	360 (4200)	300 (2520)	255 (2640)	202.5 (2520)
				
**Leisure-time PA**				
Walking	40 (840)	100 (840)	35 (600)	16.25 (840)
Vigorous PA	0 (180)	0 (120)	0 (120)	0 (600)
Moderate PA	0 (360)	0 (60)	0 (180)	0 (180)
Total	60 (840)	120 (840)	60 (600)	60 (840)
				
**Combined Domains**				
Total Walking	225 (3360)	290 (2850)	167.5 (1740)	155 (1925)
Total Moderate PA	420 (4380)	405 (2760)	360 (2640)	262.5 (2590)
Total Vigorous PA	0 (1080)	0 (1800)	0 (720)	0 (600)
Total PA	690 (6300)	840 (5415)	640 (4300)	577.5 (4270)
				
**Time Spent Sitting**				
Weekday	1500 (3750)	1200 (3900)	1500 (3450)	1500 (2850)
Weekend	480 (1320)	360 (1200)	600 (1200)	600 (1320)
Total	1980 (4650)	1680 (5100)	2130 (4170)	2100 (3630)

Values are median (*Mdn*) and range (*r*).

In the results section of the manuscript the median differences presented in the text for the Wilcoxon's signed-rank tests in the intervention group should be,

1. Leisure minutes walked: "60" not "100"

2. Weekday sitting: "- 300" not "1200"

3. Weekend sitting: "- 120" not "360"

4. Total sitting: "- 300" not "1680"

In the results section of the manuscript the median difference presented for the Mann Whitney U test at week 12 between groups should be,

1. Total minutes walked: "135" not "57.5"

As a consequence of the amendment to the median leisure minutes walked in the intervention group the following sentence from the discussion should be corrected as follows:

Original Sentence

"*The increase observed in the step-count data was supported by the self-reported results of the IPAQ; a significant increase in reported minutes of leisure time walking was found in the intervention group (median increase of 100 minutes per/week)."*

Amended Sentence

"*The increase observed in the step-count data was supported by the self-reported results of the IPAQ; a significant increase in reported minutes of leisure time walking was found in the intervention group (median increase of 60 minutes per/week)."*
